# Xylitol production from xylose mother liquor: a novel strategy that combines the use of recombinant *Bacillus subtilis *and *Candida maltosa*

**DOI:** 10.1186/1475-2859-10-5

**Published:** 2011-02-07

**Authors:** Hairong Cheng, Ben Wang, Jiyang Lv, Mingguo Jiang, Shuangjun Lin, Zixin Deng

**Affiliations:** 1 Laboratory of Microbial Metabolism and School of Life Sciences and Biotechnology, Shanghai Jiao Tong University, 800# Dongchuan Road, Shanghai, China; 2 Key Laboratory of Chemical and Biological Transforming Process, School of Chemistry and Ecology Engineering, Guangxi University for Nationalities, Nanning, China

## Abstract

**Background:**

Xylose mother liquor has high concentrations of xylose (35%-40%) as well as other sugars such as L-arabinose (10%-15%), galactose (8%-10%), glucose (8%-10%), and other minor sugars. Due to the complexity of this mother liquor, further isolation of xylose by simple method is not possible. In China, more than 50,000 metric tons of xylose mother liquor was produced in 2009, and the management of sugars like xylose that present in the low-cost liquor is a problem.

**Results:**

We designed a novel strategy in which *Bacillus subtilis *and *Candida maltosa *were combined and used to convert xylose in this mother liquor to xylitol, a product of higher value. First, the xylose mother liquor was detoxified with the yeast *C. maltosa *to remove furfural and 5-hydromethylfurfural (HMF), which are inhibitors of *B. subtilis *growth. The glucose present in the mother liquor was also depleted by this yeast, which was an added advantage because glucose causes carbon catabolite repression in *B. subtilis*. This detoxification treatment resulted in an inhibitor-free mother liquor, and the *C. maltosa *cells could be reused as biocatalysts at a later stage to reduce xylose to xylitol. In the second step, a recombinant *B. subtilis *strain with a disrupted xylose isomerase gene was constructed. The detoxified xylose mother liquor was used as the medium for recombinant *B. subtilis *cultivation, and this led to L-arabinose depletion and xylose enrichment of the medium. In the third step, the xylose was further reduced to xylitol by *C. maltosa *cells, and crystallized xylitol was obtained from this yeast transformation medium. *C. maltosa *transformation of the xylose-enriched medium resulted in xylitol with 4.25 g L^-1^·h^-1 ^volumetric productivity and 0.85 g xylitol/g xylose specific productivity.

**Conclusion:**

In this study, we developed a biological method for the purification of xylose from xylose mother liquor and subsequent preparation of xylitol by *C. maltosa*-mediated biohydrogenation of xylose.

## Background

Xylose is used to prepare xylitol by chemical hydrogenation and is purified from the acid hydrolysate of sugarcane bagasse or corncob, which are widely available in China or other countries. The acid hydrolysate of sugarcane bagasse or corncob contains xylose, L-arabinose, glucose, galactose, mannose, and other minor monosaccharides [1,2]. It also contains two major inhibitors, i.e., furfural and 5-hydroxymethylfurfural (HMF), which are the degradation products of xylose and glucose, respectively [3]. These two major inhibitors have deleterious effects on the growth of microorganisms [4,5]. The acid hydrolysate is first purified on an ion-exchange resin to decrease its conductivity to less than 20 μS/cm and is then concentrated. Xylose is crystallized and separated from the concentrated acid hydrolysate by three or more rounds of gradient cooling. This produces a hydrolysate, known as the xylose mother liquor, which is abundant, low in cost, and can be used as a feedstock. It is a viscous and reddish-brown liquor that contains 35%-40% xylose, 10%-15% L-arabinose, 8%-10% glucose, and 8%-10% D-galactose.

In China, more than 50,000 metric tons of xylose mother liquor was produced in 2009, and the management of sugars like xylose that present in the low-cost liquor is a problem. Attempts have been made to improve the value of the xylose mother liquor by individually separating xylose, L-arabinose, and galactose by simulated moving bed chromatography. However, this method is difficult to adopt due to its high running costs and equipment investments as well as its unsatisfactory and low separation efficiency. In recent times, biological removal and biotransformation have become more attractive approaches for producing high-value compounds from crude sugar feedstocks [6]. *Bacillus subtilis *is a generally regarded as safe bacterium and is used in probiotics [7]. This bacterium can grow well in medium containing xylose, L-arabinose, glucose, and galactose, and it can utilize these sugars as carbon sources. At least three genes are involved in xylose metabolism--*xylA, xylB*, and *xylR*. Together, these three genes constitute the xylose utilization operon. Xylose utilization by *B. subtilis *requires the production of *xylA *and *xylB*, which is regulated at the transcriptional level by a xylose-responsive repressor protein encoded by *xylR *and by carbon catabolite repression [8]. *B. subtilis *cannot utilize xylose when the xylose isomerase gene is disrupted by insertional inactivation, but it can still metabolize L-arabinose, galactose, and glucose. Thus, xylose is enriched in the xylose mother liquor by the selective utilization of other sugars. If yeasts are then cultured in this mother liquor, they can efficiently synthesize xylitol from it. However, *B. subtilis *cannot survive in a medium containing more than 10% xylose mother liquor due to the latter's furfural and HMF content, which can severely inhibit the growth of microorganisms by reducing cellular enzymatic biological activities, causing DNA degradation and membrane damage [5,9-11]. *Candida maltosa *ATCC28140 is capable of detoxifying furfural and HMF, and this organism grows well in a medium containing 35% xylose mother liquor. Also, this yeast can hydrogenate sugars to their corresponding sugar alcohols, for example, it can efficiently convert xylose to xylitol [12].

In this study, we designed a three-step process to biologically produce xylitol from xylose mother liquor. First, the xylose mother liquor was treated with *C. maltosa *to remove the inhibitory compounds furfural and HMF as well as glucose. The yeast cells and detoxified xylose mother liquor were recovered by centrifugation. We then constructed a genetically modified *B. subtilis *in which the xylose isomerase gene was disrupted. *B. subtilis *with the disrupted xylose isomerase gene was then cultured in the detoxified xylose mother liquor to enrich xylose; this enrichment results from the biological removal of other sugars such as L-arabinose and some of the galactose. The resulting xylose-enriched mother liquor was treated with *C. maltosa *cells to allow conversion of xylose to xylitol. After further purification by activated carbon and ion-exchange treatment, crystallized xylitol was obtained from the above-described yeast transformation medium.

## Methods

### Microorganisms and media

The strains and plasmids used in this study are listed in Table 1. Luria-Bertani (LB) agar was used to cultivate the *B. subtilis *strains, and the medium was supplemented with chloramphenicol (25 mg L^-1^) when necessary. The SP salt solution contained 0.2% (NH_4_)_2_SO_4_, 1.4% K_2_HPO_4_, 0.6% KH_2_PO_4_, 0.02% MgSO_4_·7H_2_O, and 0.1% sodium citrate. The CAYE solution (100×) consisted of 2% casamino acids and 10% bacto-yeast extract. The SPI medium (SP salt solution supplemented with 0.5% sugar and 1% 100 × CAYE) and SPII medium (SPI medium supplemented with 1% 50 mM CaCl_2 _and 1% 250 mM MgCl_2_) were used for *B. subtilis *transformation. The xylose mother liquor used in this study was purchased from Jiahe Sugar Co. Ltd.(Changyi City, Shandong Province, China), it contains 690 g L^-1 ^dissolved solids (DS) consisting of 350 g L^-1 ^xylose, 150 g L^-1 ^L-arabinose, 80 g L^-1 ^glucose, 80 g L^-1 ^galactose, and less than 30 g L^-1 ^unknown sugars (mannose, rhamnose, and oligosaccharides).

**Table 1:** Bacterial strains, yeast strain, and plasmids used in this study

Strain or plasmid	Relevant marker(s)	Source
*Bacillus subtilis *subsp. *subtilis *168	*trpC2*	BGSC 1A1
*Candida maltosa *ATCC28140		ATCC
pBlueScript II SK(-)	*Ap*^*r*^	Stratagene
pBCJ164.3	*Ap^r^Cm^r^*	BGSC ECE176
pMD-xyl	*Ap^r^*	This study
pMD-cm	*Ap^r^Cm^r^*	This study
pBS-Xyl	*Ap^r^*	This study
pBS-Xyl-Cm	*Ap^r^Cm^r^*	This study

### Plasmid construction

**Figure 1:**
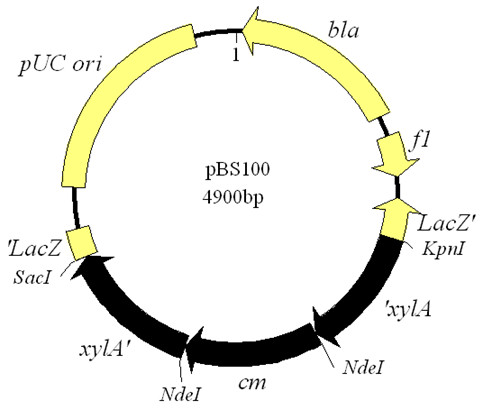
**Map of plasmid pBS100**. *'xylA*, xylose isomerase gene 5' fragment (659 bp); *xylA'*, xylose isomerase gene 3' fragment (679 bp); *'LacZ*, β-galactosidase α-fragment 5' part; *LacZ'*, β-galactosidase α-fragment 3' part; *fi*, replication origin; *cm*, chloramphenicol acetyltransferase gene; *bla*, ampicillin resistance gene.

The *xylA *gene was amplified by PCR using *PxylA*-F and *PxylA*-R as gene-specific primers and genomic DNA from *B. subtilis *subsp. *subtilis *168 as the template. The PCR fragment (*xylA *gene) was ligated to the pMD18 T-simple vector to form the recombinant plasmid pMD-xylA. The latter was digested with *Kpn*I and *Sac*I, and the fragment *Kpn*I/*xylA/Sac*I was released. This fragment was ligated with *Kpn*I- and *Sac*I-digested pBlueScript II SK(-), resulting in the recombinant plasmid pBS-*xylA*. The *cm*^r ^gene was amplified by PCR using *Pcm*-F and *Pcm*-R as gene-specific primers and plasmid pBCJ164.3 as the template. The DNA fragment (0.65 kb) was ligated to the pMD18 T-simple vector to form pMD-cm. The recombinant plasmid pMD-cm was then digested with *Nde*I. The *Nde*I/*cm*/*Nde*I fragment that was released was ligated with *Nde*I-digested pBS-*xylA*, resulting in the recombinant plasmid pBS100 (Figure 1). The *xylA *gene was disrupted by insertion of the chloramphenicol resistance gene (*cm*). All the primers used here are listed in Table 2.

**Table 2:** Sequences of the primers used in this study

Primer	Sequence	Enzyme site
*PxylA*-F	5'-tg ggt acc atg gct caa tct cat tcc agt tc-3'	*Kpn*I
*PxylA*-R	5'-tg gag ctc t tat act tct aaa atg tat tgg ttc-3'	*Sac*I
*Pcm*-F	5'-tg cat atg agg agg atg atg aac ttt aat aaa att gat tta g-3'	*Nde*I
*Pcm*-R	5'-tg cat atg tt ata aaa gcc agt cat tag gcc-3'	*Nde*I

Restriction sites are underlined.

### Bacterial transformation

The *B. subtilis *subsp. *subtilis*168 strain to be transformed was streaked on an LB agar plate and incubated at 37°C. A single colony was inoculated in 3 mL LB broth and cultured overnight. A 200-μL aliquot of the culture was transferred to 8 mL SPI medium and cultured at 37°C and 200 rpm until its OD_600 _reached approximately 0.8-1.2. Next, 200 μL of this culture was transferred to 2 mL SPII medium and cultured at 100 rpm for 90 min. EGTA (20 μL of a 10 mmol L^-1 ^solution) was added to this culture, which was left to incubate for an additional 10 min at 100 rpm. The cells were divided into 500-μL aliquots and placed in 1.5-mL Eppendorf tubes. The *Pvu*II-linearized plasmid pBS100 (5 μL) was added to 500 μl of competent cells and incubated for 90 min at 100 rpm. All the cells were then plated onto two LB agar plates supplemented with chloramphenicol (25 μg mL^-1^) and incubated at 37°C for 2 days until positive recombinants appeared.

### Identification of the *xylA *gene-disrupted positive colonies

Two transformants that grew on LB agar plates containing 25 μg mL^-1 ^chloramphenicol were selected to check whether the xylose isomerase gene was disrupted by the insertion of the chloramphenicol resistance gene. Two sets of primers, i.e., P*xylA*-F and P*cm*-R, P*cm*-F and P*xylA*-R, were used to amplify the 1310 bp and 1330 bp fragments, respectively, from the genomic DNA of the two transformants. The genomic DNA of wild-type *B. subtilis *subsp. *subtilis *168 was used as the negative control. To clarify recombinant cell extracts that had no xylose isomerase activity, recombinant (Bsxyl strain) and nonrecombinant cells were cultivated in 5 mL of liquid LB medium supplemented with 5 g L-arabinose L^-1 ^and 5 g xylose L^-1 ^for 24 h. They were then centrifuged at 10000 *g *and resuspended in 2 mL of 50 mM Tris-Cl buffer (pH 6.5). The cells were disrupted by supersonication, and clear supernatants were obtained by centrifugation. A 20-μL aliquot of the supernatant was used as the crude enzyme and reacted with 200 μL of 50 g L^-1 ^xylose in 50 mM Tris-Cl buffer (pH 6.5) at 37°C for 5 h. Subsequently, 50 μL of the reaction buffer was analyzed by HPLC to detect whether D-xylulose had formed from xylose.

To further clarify whether or not the recombinant cells could utilize xylose, recombinant and nonrecombinant *B. subtilis *subsp. *subtilis*168 cells were cultivated in LB medium containing 1.0% L-arabinose with or without chloramphenicol at 37°C and 200 rpm. The cells were collected by centrifugation and resuspended in LB broth containing 1.0% xylose, followed by culture at 37°C and 200 rpm. Samples were taken at regular intervals and subjected to high-performance TLC (using a silica gel plate purchased from Merck, Rahway, New Jersey) to determine the change in the xylose content. The silica plate was developed with a chromatography solution (pyridine:ethyl acetate:acetate:water = 5:5:3:1) in a developing tank. After completion, the plate was dried and sprayed with 1% sodium periodate. After further drying, it was sprayed with a color developing agent (1% benzidine in 95% ethanol). The xylose spots were clearly visualized by this method.

### Removal of inhibitors by *C. maltosa*

To evaluate the ability of *C. maltosa *to remove inhibitors, the cells were inoculated into synthetic dextrose medium containing 10 g L^-1 ^YNB (with histidine and ammonium sulfate, Difico™, MD, USA), 10 g L^-1 ^glucose, and furfural (5 mM, 10 mM, 20 mM, 30 mM, or 40 mM, TCI, Tokyo) or HMF (in the same range from 5 mM to 40 mM, TCI, Tokyo). In the case of furfural, 1 mL furfural was first resolved in 10 mL of 100% ethanol, and distilled water was added to prepare a 100 mM stock solution. The control was prepared without furfural and HMF. The assays were performed on a shaker at 32°C and 180 rpm for 60 h using 250-mL Erlenmeyer flasks filled with 50 mL of the above media. Samples were periodically removed every 12 h to estimate the furfural and HMF concentrations as well as cell growth.

### Fermentation profile of *C. maltosa *in the xylose mother liquor

The *C. maltosa *inoculum was prepared by transferring cells from the maintenance medium (maltose extract agar) to a 50-mL test tube containing 15 mL of YPD medium (consisting of 10 g L^-1 ^yeast extract, 5 g L^-1 ^tryptone, and 10 g L^-1 ^dextrose). Incubation was carried out at 32°C and 200 rpm for 24 h. The cells were recovered by centrifugation and transferred to a 250-mL Erlenmeyer flask containing 50 mL of medium (consisting of 15 g L^-1 ^yeast extract and 200 g L^-1 ^xylose mother liquor, pH 5.5 (autoclaved at 105°C for 15 min)). The medium was cultivated at 200 rpm and 32°C for 60 h. Samples were periodically removed at 12-h intervals, and the concentrations of glucose, L-arabitol, and xylitol were determined.

### Xylose enrichment from xylose mother liquor using *C. maltosa *and *B. subtilis *strain BSxyl

Fermentation medium (containing 15 g L^-1 ^yeast extract and 200 g L^-1 ^xylose mother liquor) was first treated with yeast *C. maltosa *cells to remove glucose and the inhibitors, as described above. Recombinant *B. subtilis *BSxyl was first cultivated in 5 mL of LB medium at 37°C and then transferred to 40 mL of detoxified fermentation medium. The culture was incubated overnight at 37°C with shaking at 200 rpm. It was then transferred to 360 mL of detoxified fermentation medium in a 2-L flask at 37°C and shaken at 200 rpm for 12 h to obtain 400 mL of second-class seed. This 400-mL seed culture was transferred to a 5-L fermentor containing 4 L of detoxified fermentation medium in order to enrich xylose by depleting L-arabinose and some of the galactose present. Samples were taken at regular intervals and analyzed by HPLC to determine the content and relative purity of xylose. Fermentation was completed when the L-arabinose in the medium was completely exhausted. The xylose-enriched clear fermentation broth was obtained by centrifugation. Yeast *C. maltosa *cells were added to the broth to synthesize xylitol, as described below.

### Model sugar studies

To evaluate the influence of the sugar components of the xylose mother liquor on the growth of *B. subtilis *strain BSxyl in the absence of inhibitors, this strain was cultured in 250-mL shake flasks containing 50 mL of the model sugars medium. This medium consisted of 10 g L^-1 ^yeast extract and the following model sugars: 70 g L^-1 ^xylose, 30 g L^-1 ^L-arabinose, 16 g L^-1 ^glucose, 16 g L^-1 ^galactose, 2 g L^-1 ^mannose, 2 g L^-1 ^rhamnose, and 2 g L^-1 ^oligoxylosaccharide. The DS content was 130 g L^-1 ^in 20% xylose mother liquor. The cells were incubated for 60 h at 37°C on a rotary shaker at 200 rpm.

### Reduction of xylose to xylitol by transformation with the yeast *C. maltosa*

The xylose-enriched clear supernatant, obtained as described above, was concentrated to a xylose content of 250 g L^-1 ^(w/v). Different concentrations of *C. maltosa*, ranging from 30 to 60 g L^-1 ^dry weight of cells (1 mL cell suspension with an OD_600 _value of 1 was equivalent to 0.30 mg dry weight of *C. maltosa*) and obtained from the stage after the removal of inhibitors, were suspended in the xylose concentrate and used to reduce xylose to xylitol. Samples were withdrawn at regular intervals and assayed. After the reduction of xylose to xylitol, a clear transformation broth was obtained by centrifugation, and it was treated with ion-exchange resins to reduce its conductivity to less than 100 μs cm^-1^. Activated charcoal was used to obtain a colorless solution, which was then concentrated by rotary vacuum evaporation at 65°C. This increased the xylitol content to 800 g L^-1^.

### Preparation of xylitol crystals

Xylitol seed was added to the colorless solution containing 800 g L^-1 ^xylitol at 65°C to increase the supersaturation, and the solution was then transferred to a 10-L crystallizer that was subjected to linear cooling from 65°C to 15°C in 50 h (one degree decrease per hour) with simultaneous (periodic) stirring. Xylitol crystals were separated from the crystallization mass by centrifugation. The resulting xylitol mother liquor was further concentrated to 800 g L^-1 ^by rotary vacuum evaporation at 65°C and then subjected to crystallization under the same conditions. Generally, xylitol can be crystallized three times from the above purified colorless solution.

### Analytical methods

The concentrations of sugar and sugar alcohols were determined using an HPLC system equipped with a Shodex RI 101 refractive index detector and an analytical Shodex SPO 810 sugar column (8 × 30 mm, Pb^2+ ^cation exchange column). A sample (50 μL) was injected into the HPLC system and eluted with distilled water at a column temperature of 70°C and a flow rate of 1.2 mL min^-1^. The absorbance at 280 nm (*A*_280_) was an indicator of the concentration of the inhibitors (furfural or HMF) in the medium because these inhibitors have maximum absorbance at 280 nm.

## Results and discussion

### Identification of the recombinant *B. subtilis *strain BSxyl

**Figure 2:**
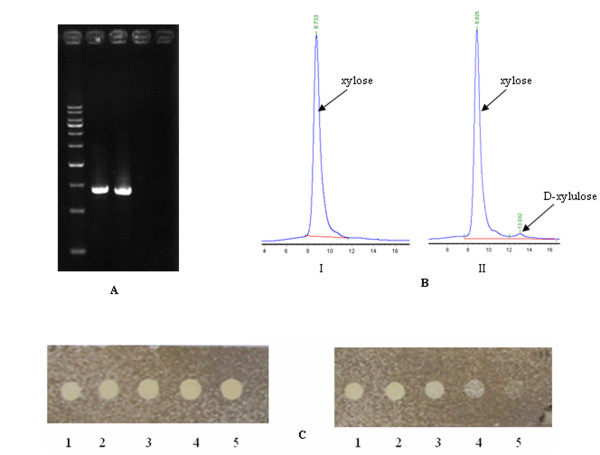
**Identification of recombinant B. subtilis carrying the xylose isomerase gene disruption**. A, PCR identification of two recombinant *B. subtilis *strains carrying the xylose isomerase gene disruption (lanes 1 and 2) and two wild-type *B. subtilis *strains (lanes 3 and 4). To create the recombinant strains, the 1.4-kb DNA was amplified using two sets of primers (see Method and Materials); B, HPLC analysis of the xylose isomeric reaction. The xylose isomeric reaction with the crude extract from strain BSxyl (*xylA *disrupted); DNA markers from the bottom of the gel are in the following order: 500 bp, 1 kb, 1.5 kb, and 2 kb. B, HPLC analysis of the xylose isomeric reaction with the crude extract from recombinant (I) and wild-type *B. subtilis *168 (II). No D-xylulose was detected after incubation with the crude extract from the *xylA-*gene-disrupted strain BSxyl (I), while D-xylulose could be detected using the crude extract from the control strain *B. subtilis *168 as enzyme and xylose as substrate (II). C, TLC assay of the culture medium of recombinant BSxyl (i) and *B. subtilis *168 (ii). The culture medium consisted of LB and 10 g L^-1 ^xylose. The spot in 1 represents 1 μL of 10 g L^-1 ^xylose standard, and the spots in 2, 3, 4, and 5 show 1 μL of each sample taken at 0, 8, 16, and 24 h.

To determine whether the *Cm *gene was inserted into the chromosome of *B. subtilis *strain BSxyl at the xylose isomerase gene locus, two sets of primers were used to amplify the corresponding DNA with genomic DNA as the template. Two DNA fragments of size 1.4 kb were amplified with the two sets of primers and the recombinant strain *B. subtilis *BSxyl genomic DNA, whereas no DNA bands were amplified when the nonrecombinant strain's genomic DNA was used as the template (Figure 2A). This indicated that the *Cm *gene was inserted at the locus of the xylose isomerase gene.

To determine whether the xylose isomerase gene was inactivated by *Cm *gene insertion, xylose-to-xylulose enzymatic conversion assays were conducted using crude cell extracts as the enzyme solution. The HPLC analysis (Figure 2B) revealed that strain BSxyl did not have any active xylose isomerase and was therefore unable to transform xylose to D-xylulose. In contrast, the crude extract from nonrecombinant *B. subtilis *168 could convert xylose to D-xylulose.

Additionally, the recombinant *B. subtilis *strain BSxyl and nonrecombinant one were cultured in LB medium containing 10 g L^-1 ^xylose, as described in Methods and Materials. Throughout the 24-h cultivation period, the xylose content remained almost constant for *B. subtilis *BSxyl, while it decreased in the control strain *B. subtilis *168, indicating that strain BSxyl was almost unable to utilize xylose after disruption of the xylose isomerase gene. After incubation for 24 h, the xylose content of the BSxyl recombinant strain culture was 9.24 g L^-1^, as calculated by the integration of the HPLC peaks, while that of the control strain was 3.45 g L^-1^. The TLC assay further proved that the recombinant *B. subtilis *strain BSxyl could not utilize xylose. When samples taken from the medium at 0, 8, 16, and 24 h were applied to the silica gel plate, the brightness of the spot for strain BSxyl remained unchanged, while that of the spot for the control strain decreased (Figure 2C). Thus, the genetically modified *B. subtilis *strain in which the xylose isomerase gene was disrupted could not utilize xylose but could utilize glucose, L-arabinose and to a lesser extent galactose. Galactose is not actively transported into *B. subtilis *168. Even low amounts of internal UDP-galactose are toxic for *galE*-negative *B. subtilis *strains [13]. *B. subtilis *168 cannot grow on galactose or xylose as the sole carbon source due to its inability to transport these sugars from the outside to the inside of the cell [14]. However, *B. subtilis *can use D-galactose and D-xylose as carbon sources when L-arabinose, the inducer of monosaccharide transporter protein (AraE) synthesis, is added to the growth medium [15,16]. *B. subtilis *can also grow on L-arabinose as the sole carbon and energy source. The ability to utilize L-arabinose depends on the presence of three intracellular enzymes encoded by the *ara*A (L-arabinose isomerase), *ara*B (L-ribulokinase), and *ara*D (L-ribulose-5-phosphate-4-epimerase) genes, which sequentially convert L-arabinose to L-ribulose, L-ribulose-5-phosphate, and D-xylulose-5-phosphate, respectively. D-Xylulose-5-phosphate is further catabolized through the pentose phosphate pathway [17]. BSxyl first utilizes glucose and then utilizes L-arabinose which then induces the AraE protein to transport xylose and galactose into the cell cytoplasm.

The xylose mother liquor contains 150 g L^-1 ^L-arabinose as well as 350-400 g L^-1 ^xylose. Although *C. maltosa *used in this study could spontaneously and efficiently reduce xylose and L-arabinose to xylitol and L-arabitol, it is very difficult to separate xylitol due to the presence of L-arabitol which greatly reduces the crystallization efficiency of xylitol. An alternative method would be to remove L-arabinose from the xylose mother liquor, prior to the latter's use as a raw material for the reduction of xylose to xylitol by yeast cells. Recombinant *B. subtilis *BSxyl could utilize L-arabinose but not xylose; therefore, these cells could be used to deplete L-arabinose and enrich xylose in the xylose mother liquor. As a result, L-arabitol was not produced during yeast transformation using the xylose mother liquor as the raw material; this facilitated the crystallization of xylitol and improved the purity of the xylitol obtained from the yeast transformation medium.

### Removal of inhibitors by *C. maltosa*

It has been shown that furfural and HMF have maximum absorbance at 280 nm (*A*_280_). Since no other proteins were detected in the xylose mother liquor, the *A*_280 _value could be used as an indicator of the content of furfural and HMF [18]. These two compounds are the main inhibitors present in the xylose mother liquor. We obtained the following relationship between the furfural or HMF concentration and the *A*_280 _value: C_(furfural) _= 0.732 + 60.49 × *A*_280_, C_(HMF) _= 0.603 + 49.62 × *A*_280_. The R^2 ^value was 0.9994 and 0.9997, respectively. C represents the concentration of furfural or HMF (μM), and *A*_280 _is the absorbance at 280 nm. A 100-μL aliquot of *C. maltosa *cells in the exponential phase cultivated in YPD medium was transferred to 25 mL of fresh YNB medium containing various concentrations of furfural or HMF (see Method and Material). Each culture was inoculated with the same cell concentration (OD_600 _at 0.13), and the ability of the cells to grow was determined by monitoring the cell densities at OD_600 _over a period of 60 h. The *A*_280 _was also monitored to determine the concentration of total inhibitors (furfural and HMF). As shown in Table 3, no significant decrease in growth was observed for *C. maltosa *cultivated in medium containing 5-20 mM furfural or HMF over 60 h. In comparison to cells cultivated in medium without furfural or HMF, the growth of *C. maltosa *was inhibited by about 25% and 50% in the presence of 30 mM furfural or 40 mM HMF, respectively, over a period of 60 h. No significant decrease in growth was observed at furfural or HMF concentrations less than 20 mM; consequently, no differences were observed in the lag times and doubling times of *C. maltosa*. However, in the presence of 30 mM furfural or 40 mM HMF, the lag times and doubling times were longer (data not shown). The ability of *C. maltosa *to metabolize furfural or HMF was also tested by monitoring the *A*_280_. The *A*_280 _value decreased to less than 0.05 from 81.4, 162.8, and 325.6, which corresponded to 5, 10, and 20 mM of furfural or HMF, respectively, over a period of 36 h. This indicated that *C. maltosa *could metabolize furfural or HMF in 36 h. Although *C. maltosa *showed longer lag times and doubling times as well as 25%-50% growth inhibition at 30 mM and 40 mM furfural or HMF, the *A*_280 _value also decreased to less than 0.05 from 488.4 and 651.2 after 60 h of cultivation. These results indicated that yeast *C. maltosa *can efficiently metabolize inhibitors such as furfural or HMF. The total concentration of furfural and HMF in the xylose mother liquor was approximately 40 mM, as calculated from the above formula. Thus, *C. maltosa *could be used to detoxify the xylose mother liquor and remove inhibitors such as furfural and HMF, which were formed by the dehydration of released sugars (xylose and glucose) from corncobs or sugarcanes pretreated with dilute acid. The *A*_280 _value also decreased to less than 0.05 from 135.6 when *C. maltosa *cells were cultivated in medium containing 200 g L^-1 ^xylose mother liquor and 20 g L^-1 ^YNB for 36 h at 33°C and 200 rpm. Interestingly, no xylitol accumulation was observed during the bio-detoxification and glucose depletion process (data not shown). There are many literatures reported that furfural or HMF in undetoxified lignocellulose could act as external electron acceptors, thus repress the xylitol production in lignocellulose fermentation by yeast strains [19,20]. For corncob hydrolysate which contains less furfural than softwood hydrolysates, xylitol could be accumulated in the fermentation medium [21]. Whereas xylose mother liquor used in this study contains about 40 mM furfural and HMF, which could inhibit xylitol formation during the first treatment by *C. maltosa*. The mechanism by which furfural and HMF are degraded in *Saccharomyces cerevisiae *has been clarified in detail and involves multiple genes including those encoding NAD(P)H-dependent aldehyde reductases and alcohol dehydrogenases which reduce furfural and HMF to less cytotoxic alcohols [22-24]. More recently, the alcohol dehydrogenase genes (*CmADHs*) from *C. maltosa *were cloned and were functionally identified to play a critical role to maintain NADH/NAD ratio, which might closely relate to detoxifying furfural and HMF [12,22]. During the biodetoxification of xylose mother liquor by *C. maltosa*, NAD(P)H produced by depletion of glucose might be acted as cofactors to reduce furfural and HMF to alchols but not reduce xylose to xylitol, resulting no xylitol production during the first treatment with *C. maltosa*. Whether the degradation mechanism in *C. maltosa *is the same as that in *S. cerevisiae *is still under investigation.

**Table 3:** The growth profile (OD_600_) and removal of inhibitors (*A*_280_) of *C. maltosa *from YNB medium containing various concentrations of furfural or HMF

Conc. (mM)		Furfural							HMF					
		
		0 h	12h	24h	36h	48h	60h		0h	12h	24h	36h	48h	60h
0	OD_600_	0.13	3.35	7.32	12.4	14.6	16.2		0.13	3.76	8.96	13.4	15.4	16.6
	*A*_280_	0.003	0.002	0.003	0.003	0.002	0.003		0.002	0.002	0.002	0.003	0.002	0.002
5	OD_600_	0.13	3.42	7.89	13.6	15.6	17.6		0.13	3.45	7.98	13.6	14.8	16.8
	*A*_280_	81.4	35.6	0.05	0.006				81.4	40.2	0.09	0.004		
10	OD_600_	0.13	3.25	7.22	12.6	14.4	16.0		0.13	3.24	7.26	12.4	13.6	15.8
	*A*_280_	162.8	122.4	62.4	0.005				162.8	132.4	60.6	0.005		
20	OD_600_	0.13	3.02	6.86	12.0	13.2	15.4		0.13	3.12	6.82	11.2	12.6	14.6
	*A*_280_	325.6	290.2	128.4	0.05				325.6	270.6	110.4	0.05		
30	OD_600_	0.13	1.86	5.20	9.68	10.6	12.6		0.13	1.68	4.94	8.86	9.84	11.8
	*A*_280_	488.4	440	363.2	220.6	120.3	0.05		488.4	405.6	368.4	282.4	144.4	0.05
40	OD_600_	0.13	0.78	2.66	4.24	6.64	8.60		0.13	0.86	3.06	5.46	7.14	8.86
	*A*_280_	651.2	545.2	410.2	330.4	150.6	0.05		651.2	520.6	443.6	308.2	163.4	0.05

For details, see Methods and Materials. Values are the means of two dependent experiments.

### Enrichment of xylose by *C. maltosa *and *B. subtilis *BSxyl

**Figure 3:**
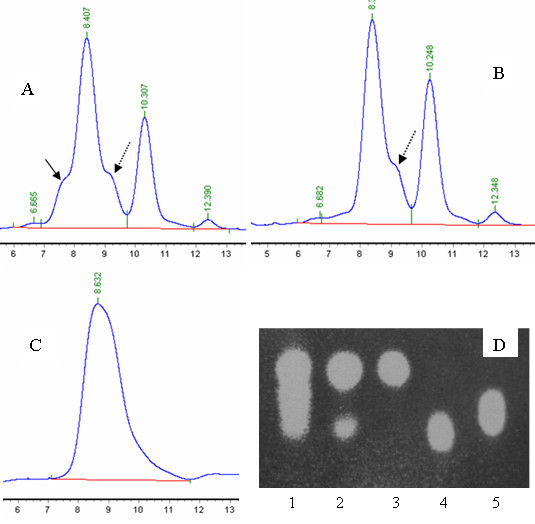
**HPLC and TLC analyses of the xylose mother liquor treated with *C. maltosa *and *B. subtilis *strain BSxyl**. A, xylose mother liquor containing glucose (showed by solid line), xylose (8.407 min), galactose (showed by dotted line), L-arabinose (10.307 min), and unknown sugars (6.665 min and 12.390 min); B, xylose mother liquor treated with *C. maltosa *for 12 h (glucose is depleted); C, *B. subtilis *strain BSxyl was inoculated in xylose mother liquor pretreated with *C. maltosa *for 60 h. L-arabinose as well as some galactose and unknown sugars (6.682 min and 12.348 min) were depleted. D, TLC analysis of the *B. subtilis *strain BSxyl final fermentation medium (C) to detect whether galactose is still present. Lane 1, xylose mother liquor; lane 2, *B. subtilis *strain BSxyl final fermentation medium (C); lanes 3 to5, xylose, galactose, and L-arabinose standards, respectively.

*B. subtilis *strain BSxyl showed poor growth when it was inoculated into a fermentation medium containing 20% xylose mother liquor and 1.5% yeast extract and cultured at 37°C and 200 rpm. This is probably due to the high amounts of inhibitory compounds (8 mM furfural and HMF in 20% xylose mother liquor) present in the culture medium. In contrast, this microorganism grew well in the same fermentation medium detoxified with *C. maltosa *and in the model sugar medium (data not shown). These results indicated that furfural and HMF present in the xylose mother liquor can inhibit the growth of *B. subtilis *strain BSxyl. Moreover, *C. maltosa *could degrade these inhibitory compounds and exhaust all the glucose in the xylose mother liquor (Figure 3, A and 3B), thereby eliminating the carbon catabolite repression of *B. subtilis *[25] and accelerating the metabolism of L-arabinose. After detoxification of the medium by *C. maltosa*, *B. subtilis *strain BSxyl grew well in the fermentation medium containing 20% xylose mother liquor and 1.5% yeast extract. The L-arabinose and galactose contents decreased with the fermentation time, while the xylose content remained constant during fermentation. After 60 h of cultivation, L-arabinose was completely depleted (Figure 3, C), while the xylose content (70 g/L of xylose) remained almost the same as that at the beginning of fermentation. Due to the strain's poor ability to utilize galactose, a small amount of residual galactose was also detected in the fermentation medium after fermentation (Figure 3, D, lane 2) by TLC analysis. The purity of xylose plus trace amounts of D-galactose in the fermentation broth was as high as 85% (data not shown).

### Biological synthesis of xylitol from the xylose-enriched fermentation broth using *C. maltosa*

**Figure 4:**
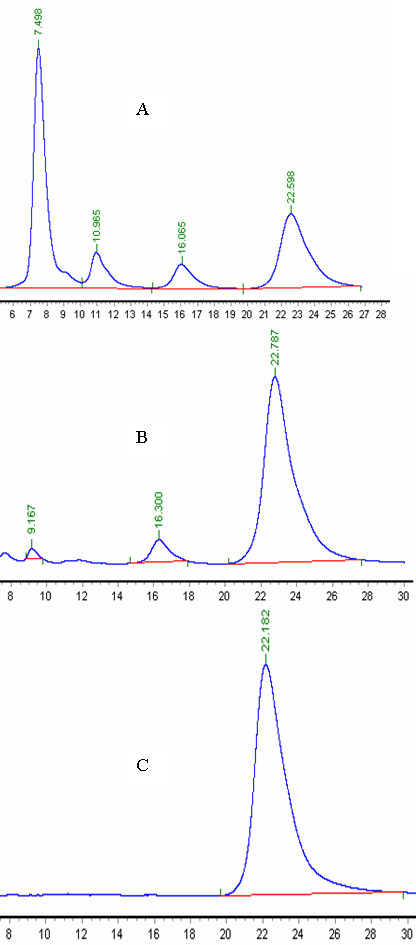
**HPLC analysis of the biotransformation of xylose to xylitol by *C. maltosa *and of crystallized xylitol from the biotransformation broth**. A, biotransformation of xylose to xylitol by *C. maltosa *for 24 h; B, 50 h; C, crystallized xylitol from the biotransformation broth. Vertical arrow in A at 9.167 min represents ethanol, and that at 10.965 min in A indicates glycerol. Similarly, the arrow at 16.065 min in A and 16.300 min in B represent D-arabitol and that at 22 min in A, B, and C indicates xylitol.

**Figure 5:**
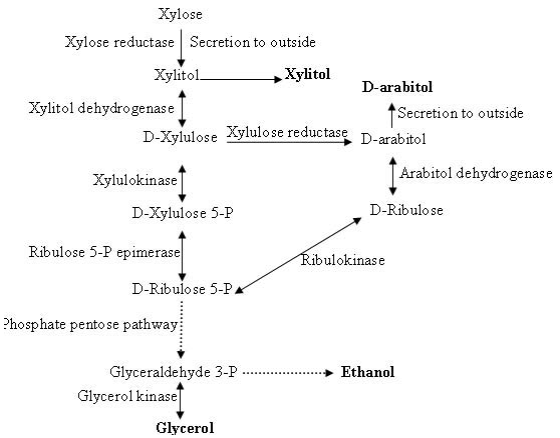
**Possible pathways for the conversion of xylose to D-arabitol, glycerol, and ethanol by *C. maltosa***. Dotted arrow shows multiple reaction steps.

Microbial reduction of the xylose-enriched broth by *B. subtilis *BSxyl was performed using *C. maltosa *cells as the biocatalyst, and 213 g L^-1 ^xylitol was obtained from the medium containing 250 g L^-1 ^xylose over a period of 50 h. The volumetric productivity of xylitol was 4.25 g L^-1 ^·h^-1^, and the conversion yield was 0.85 g xylitol per g xylose. The volumetric productivity of xylitol was shown to be closely related to the cell concentration, with increased productivity resulting from an increased initial cell concentration [26]. The volumetric productivity and specific productivity of xylitol were 4.25 g L^-1 ^h^-1 ^and 0.85 g xylitol per g xylose, respectively, over 50 h when 30 g L^-1 ^dry weight of cells was used at 1.0 vvm aeration and 200 rpm stirrer speed in a 5-L fermentor. The fermentation time was reduced to 28 h by increasing the initial cell concentration to 50 g L^-1 ^in the same transformation medium and under the same conditions. The volumetric productivity was 7.5 g L^-1^·h^-1^, and the specific productivity was 0.82 g xylitol per g xylose. From the results, we concluded that higher volumetric productivity was achieved when a higher cell concentration was used. Although the volumetric productivity increased, the specific productivity remained almost constant, regardless of the initial cell concentration. Interestingly, in addition to glycerol and ethanol, the yeast produced D-arabitol as a by-product from xylose (Figure 4, A and 4B). The glycerol level rapidly decreased throughout the culture period, from 7.25 g L^-1 ^at 24 h to less than 1 g L^-1 ^at 50 h, but the D-arabitol and ethanol levels decreased very slowly, from 5.26 g L^-1 ^and 2.42 g L^-1 ^at 24 h to 5.02 g L^-1 ^and 2.34 g L^-1 ^at 50 h, respectively. Figure 5 shows a possible pathway for the conversion of xylose to D-arabitol, glycerol, and ethanol. D-arabitol was secreted into the medium and could be detected by HPLC when xylitol (ranging from 50 to 200 g L^-1^) was used as the substrate. However, no xylitol was detected in the medium when D-arabitol was used as the substrate (50-200 g L^-1^) (data not shown). These results indicate that D-xylulose was reduced to D-arabitol by D-xylulose reductase, which catalyzes the irreversible reaction from D-xylulose to D-arabitol. On the other hand, D-arabitol was oxidized to D-ribulose by D-arabitol dehydrogenase and was converted to D-ribulose-5-P, which then entered the pentose phosphate pathway. Xylitol was crystallized from the purified xylitol-enriched solution, and its purity reached 99.5%. No D-arabitol was detected by HPLC analysis (Figure 4, C).

Recently, *Candida *yeasts, particularly *C. tropicalis*, *C. guilliermondii*, and *C. parapsilosis*, have been extensively studied for their possible applications to the production of xylitol from pure xylose or xylose-containing hydrolysates such as sugarcane bagasse or corn cob hydrolysates [4,18,27]. Some of their favorable properties include their high xylitol yields from xylose [28] and their excellent adaptability [4,29]. Although *C. tropicalis*, *C. guilliermondii*, and *C. parapsilosis *could grow on media containing high concentrations of pure xylose (250 g L^-1^) and produce xylitol with specific productivity ranging from 0.65 to 0.78 g/g xylose, these three yeasts grew poorly in media containing 300 g L^-1 ^xylose mother liquor and furfural was showed to be a strong inhibitor of their growth even at a concentration of 0.2% [30,31]. However, *C. maltosa *used in this study could grow well on the same medium (data not shown). Thus, *C. maltosa *showed the best tolerance to high concentrations of the xylose mother liquor. Based on these properties, *C. maltosa *was selected in this study to remove the inhibitory compounds furfural and HMF as well as glucose from xylose mother liquor. L-arabinose was subsequently removed by *B. subtilis *Bsxyl, leading to the enrichment of xylose, which was then reduced to xylitol by *C. maltosa *cells. The *C. maltosa *cells used in the last step were the same as those used in the first step for detoxification and removal of glucose from the xylose mother liquor.

Large quantities of *C. maltosa *cells were obtained during the detoxification and glucose removal process, and these could then be used as biocatalysts to efficiently reduce xylose to xylitol at high volumetric productivity (4.25 g L^-1 ^h^-1 ^to 7.5 g L^-1 ^h^-1^, depending on the initial cell concentration) and 0.85 g xylitol/g xylose specific productivity. The yeast cells could be reused as biocatalysts, to reduce xylose to xylitol. Moreover, no tedious procedure was required to obtain the cells. Recently, many different yeast strains including their derivative recombinants were used as whole-cell biocatalysis to convert substrates to their products. Employing yeast strains as whole-cell biocatalysts includes many advantages: Cofactors necessary for biocatalysis can be regenerated from cheap co-substrates (e.g. glucose) and are more stable within their natural cellular environment, whole cells can be employed for repetitive biocatalysis [32]. In this study, *C. maltosa *could be employed for at least 5 times without significant decrease in their efficiency and viability (data not shown). To our knowledge, this is the first attempt to apply this biotechnique for the production of xylitol from xylose mother liquor, which is a viscous and compositionally complex low-value by-product of the xylose production industry. Further studies on the feasibility of this novel strategy for the scaled-up preparation of xylitol from xylose mother liquor are currently underway.

## Conclusions

The present work describes a novel strategy to efficiently purify and isolate pure crystallized xylitol, a more high-valued product, from low-cost material xylose mother liquor, which was sequentially treated with *C. maltosa*, *B. subtilis *Bsxyl, and *C. maltosa *again. Approximately, 70 g L^-1 ^xylose of purity degree greater than 90% could be obtained from the initial fermentation medium containing 200 g xylose mother liquor (0.35 g xylose per g mother liquor) per liter medium after the removal of glucose and L-arabinose by *C. maltosa *and *B. subtilis *Bsxyl. In a 5-L fermentor using *C. maltosa *as whole-cell biocatalyst, the specific productivity was 0.82-0.85 g xylitol per g xylose, with varying volumetric productivity depending on cell concentration. 213 g L^-1 ^xylitol was obtained from the medium containing 250 g L^-1 ^xylose bio-purified from xylose mother liquor over a period of 50 h.

## Abbreviations

Ap^r^: ampicillin resistance; Cm^r^: chloramphenicol resistance; trpC2: auxotrophic mutant of tryptophan synthesis; BGSC: Bacillus Genetic Stock Center, Ohio State University; TLC: thin-layer chromatography; HMF: 5-hydromethylfurfural; OD: optical density; xylA: xylose isomerase gene; xylB: xylulose kinase gene; xylR: regulator gene of xylose metabolism operon; EGTA: ethylene glycol bis(2-aminoethyl) tetraacetic acid; ATCC: America Type Collection Center; NADPH: nicotinamide adenine dinucleotide phosphate hydrogen; HPLC: high performance liquid chromatography; galE: UDP-galactose 4-epimerase; YNB: yeast nitrogen base; A_280_: absorbance at 280 nm.

## Authors' contributions

HRC and MGJ designed research; HRC and BW performed the plasmid construction and sugars and alcohols HPLC analysis; JYL performed the furfural and HMF degradation and fermentation experiments; SJL and ZXD provided advice on organizing the manuscript and on editorial quality; HRC and MGJ performed the literature review and drafted the manuscript. All authors have read and approved the final version of the manuscript.

## Competing interests

The authors declare that they have no competing interests.
